# Supplementary material for: Viral communities from long-term anaerobic alkane-oxidizing enrichment cultures encode predicted cell surface adhesion functions
Source: ISME J. 2026 Jul 1;20(1):wrag172. doi: 10.1093/ismejo/wrag172 (PMC13452462; doi:10.1093/ismejo/wrag172)
Supplement: Supplementary file 1 [file narayanan_etal_supplement_final_wrag172.docx]

## Supplementary information

| **Sample** | **Sampling Lat, Long** | **Incubation Temp. (˚C)** | **Energy substrate** | **Year of collection** | **Year of incubation start** | **Supplementary references for media recipe** |
| --- | --- | --- | --- | --- | --- | --- |
| Santa Monica 3A | 33.78888889, -118.6680556 | 10 | 0.2MPa Methane | 2013 | 2013 | [1] |
| Santa Monica 3B | 33.78888889, -118.6680556 | 10 | 0.2MPa Methane | 2013 | 2013 | [1] |
| Santa Monica 3C | 33.78888889, -118.6680556 | 10 | 0.2MPa Methane | 2013 | 2013 | [1] |
| Santa Monica 4A | 33.78888889, -118.6680556 | 10 | 0.2MPa Methane | 2013 | 2013 | [1] |
| Santa Monica 4B | 33.78888889, -118.6680556 | 10 | 0.2MPa Methane | 2013 | 2013 | [1] |
| Santa Monica 4D | 33.78888889, -118.6680556 | 10 | 0.2MPa Methane | 2013 | 2013 | [1] |
| Guaymas Basin Hydrogen 1A | 27.01213889, -111.4152222 | 60 | 0.2MPa 80:20 Hydrogen:CO_2_ | 2009 | 2012 | [2], Extended Methods from this study |
| Guaymas Basin Hydrogen 3 | 27.01213889, -111.4152222 | 60 | 0.2MPa 80:20 Hydrogen:CO_2_ | 2009 | 2012 | [2], Extended Methods from this study |
| Guaymas Basin Methane 1G | 27.01666667, -111.6833333 | 50 | 0.2MPa Methane | 2009 | 2010 | [3], Extended Methods from this study |
| Guaymas Basin Methane G | 27.01666667, -111.6833333 | 50 | 0.2MPa Methane | 2009 | 2010 | [3], Extended Methods from this study |
| Guaymas Basin Methane 4 | 27.01666667, -111.6833333 | 50 | 0.2MPa Methane | 2009 | 2010 | [3], Extended Methods from this study |
| Guaymas Basin Propane | 27.01213889, -111.4152222 | 50 | 0.15MPa Propane | 2009 | 2010 | [4] |
| Guaymas Basin Butane | 27.01213889, -111.4152222 | 50 | 0.15MPa Butane | 2009 | 2010 | [4] |
| Elba Seep | 42.73507778, 10.2 | 20 | 0.2MPa Methane | 2010 | 2010 | [5] |
| Menes Caldera 2 | 32.30091667, 28.43519444 | Room temperature | 0.2MPa Methane | 2003 | 2003 | [6] |
| Menes Caldera 2A | 32.30091667, 28.43519444 | Room temperature | 0.2MPa Methane | 2003 | 2003 | [6] |
| Black Sea | 44.77777778, 31.97222222 | 12 | 0.2MPa Methane | 2000 | 2004 | [6] |

**Table S1.** Incubation bottle metadata. All bottles include sulfate as an electron acceptor. A more detailed description of how the enrichment cultures were set up is contained the Supplementary Methods.

| **Sample** | **Organism (Family level)** | **Relative Abundance (Family level)** | **Number of ASVs** | **ASVs comprising >1% of reads in the sample (% total reads in sample)** |
| --- | --- | --- | --- | --- |
| Santa Monica 3A | Fermentibacteraceae | 17.4% | 13 | ASV_16 (13.6%), ASV_15 (1.4%) |
|  | ANME-2a-2b | 16.8% | 17 | ASV_10 (14.7%) |
|  | Fusibacteraceae | 16.5% | 7 | ASV_40 (14.1%) |
|  | ANME-2c | 9.9% | 2 | ASV_12 (8.1%), ASV_81 (1.8%) |
|  | Dissulfuribacteraceae | 6.0% | 8 | ASV_25 (4.1%) |
|  | Lentimicrobiaceae | 5.3% | 13 | ASV_7 (4.8%) |
|  | AKAU3564 sediment group | 5.0% | 20 | ASV_193 (1.6%), ASV_224 (1.2%) |
|  | Marinilabiliaceae | 4.7% | 6 | ASV_50 (3.4%) |
| Santa Monica 3B | ANME-2a-2b | 27.1% | 9 | ASV_10 (19.8%), ASV_6 (6.1%) |
|  | OM190 | 18.9% | 5 | ASV_32 (11.8%), ASV_22 (2.5%), ASV_124 (2.1%), ASV_128 (2.0%) |
|  | Marinilabiliaceae | 11.3% | 10 | ASV_43 (7.2%), ASV_50 (3.4%) |
|  | JS1 | 9.2% | 7 | ASV_14 (5.4%), ASV_58 (2.8%) |
|  | Fermentibacteraceae | 8.5% | 7 | ASV_15 (5.8%), ASV_31 (2.3%) |
|  | Desulfosarcinaceae | 6.6% | 18 | ASV_45 (3.3%), ASV_147 (1.2%) |
| Santa Monica 3C | Lentimicrobiaceae | 45.0% | 30 | ASV_7 (43.9%) |
|  | ANME-2a-2b | 26.3% | 6 | ASV_6 (25.7%) |
|  | Fermentibacteraceae | 10.6% | 11 | ASV_15 (9.7%) |
|  | Anaerolineaceae | 3.1% | 12 | ASV_86 (2.9%) |
|  | Desulfosarcinaceae | 3.1% | 18 | ASV_45 (1.9%) |
| Santa Monica 4A | ANME-2c | 31.0% | 7 | ASV_12 (16.7%), ASV_74 (6.7%), ASV_77 (6.3%) |
|  | Fermentibacteraceae | 9.8% | 7 | ASV_31 (9.6%) |
|  | OM190 | 9.7% | 5 | ASV_22 (8.9%) |
|  | Unclassified Methanosarciniales | 8.4% | 8 | ASV_57 (8.2%) |
|  | Marine Benthic Group D and DHVEG-1 | 5.7% | 11 | ASV_49 (4.0%) |
|  | Lokiarchaeia | 3.7% | 11 | ASV_60 (3.5%) |
|  | Desulfosarcinaceae | 3.6% | 10 | ASV_114 (2.2%) |
|  | Dissulfuribacteraceae | 3.6% | 4 | ASV_25 (2.3%) |
|  | Unclassified Phycisphaerae | 3.2% | 6 | ASV_110 (2.8%) |
| Santa Monica 4B | Dissulfuribacteraceae | 19.0% | 6 | ASV_25 (8.7%), ASV_27 (3.6%), ASV_79 (3.4%), ASV_82 (3.2%) |
|  | ANME-2c | 18.0% | 5 | ASV_12 (15.5%), ASV_81 (2.1%) |
|  | Fermentibacteraceae | 13.0% | 17 | ASV_16 (11.8%) |
|  | ANME-2a-2b | 8.5% | 21 | ASV_10 (3.7%), ASV_6 (3.4%) |
|  | OM190 | 6.5% | 4 | ASV_22 (5.4%) |
|  | Lentimicrobiaceae | 5.5% | 19 | ASV_7 (1.2%) |
|  | Marine Benthic Group D and DHVEG-1 | 3.8% | 22 | ASV_49 (1.3%) |
|  | JS1 | 3.6% | 5 | ASV_14 (2.9%) |
| Santa Monica 4D | Dissulfuribacteraceae | 18.4% | 8 | ASV_25 (5.8%), ASV_27 (5.7%), ASV_79 (3.5%), ASV_82 (3.3%) |
|  | ANME-2c | 11.6% | 5 | ASV_12 (9.0%), ASV_81 (1.7%) |
|  | ANME-2a-2b | 10.4% | 17 | ASV_10 (5.0%), ASV_6 (3.3%) |
|  | Marine Benthic Group D and DHVEG-1 | 9.3% | 21 | ASV_49 (2.8%), ASV_28 (1.2%) |
|  | Fermentibacteraceae | 8.2% | 11 | ASV_16 (7.5%) |
|  | Lentimicrobiaceae | 8.0% | 21 | ASV_7 (2.8%), ASV_164 (1.0%) |
|  | OM190 | 5.7% | 4 | ASV_22 (4.8%) |
|  | JS1 | 4.1% | 5 | ASV_14 (3.3%) |
|  | AKAU3564 sediment group | 3.3% | 17 | None (all ASVs <1%) |
| Menes Caldera 2 | Lentimicrobiaceae | 28.7% | 7 | ASV_17 (27.6%) |
|  | Marine Benthic Group D and DHVEG-1 | 25.6% | 27 | ASV_28 (9.2%), ASV_69 (6.4%), ASV_80 (4.0%), ASV_67 (2.0%), ASV_89 (1.6%) |
|  | Fermentibacteraceae | 7.2% | 4 | ASV_39 (7.1%) |
|  | Anaerolineaceae | 5.0% | 22 | ASV_64 (2.9%) |
|  | Dissulfuribacteraceae | 4.8% | 7 | ASV_87 (4.2%) |
|  | JS1 | 4.4% | 5 | ASV_14 (3.7%) |
|  | Calditrichaceae | 3.5% | 6 | ASV_41 (1.5%), ASV_150 (1.2%) |
| Menes Caldera 2A | ANME-2a-2b | 25.7% | 9 | ASV_36 (15.6%), ASV_10 (4.7%), ASV_100 (3.2%), ASV_184 (1.3%) |
|  | Marine Benthic Group D and DHVEG-1 | 21.2% | 26 | ASV_28 (7.2%), ASV_67 (3.1%), ASV_89 (2.3%), ASV_80 (2.2%), ASV_131 (1.3%), ASV_49 (1.1%) |
|  | Unclassified Plactomycetota | 9.3% | 3 | ASV_68 (8.6%) |
|  | Lentimicrobiaceae | 9.0% | 3 | ASV_85 (5.2%), ASV_17 (3.8%) |
|  | Fermentibacteraceae | 7.4% | 4 | ASV_39 (7.4%) |
|  | Anaerolineaceae | 7.3% | 18 | ASV_64 (6.4%) |
|  | Calditrichaceae | 5.5% | 5 | ASV_41 (5.1%) |
|  | JS1 | 4.4% | 4 | ASV_14 (4.2%) |
| Black Sea | JS1 | 23.0% | 14 | ASV_24 (17.1%), ASV_14 (3.7%) |
|  | Desulfosarcinaceae | 18.7% | 21 | ASV_23 (18.4%) |
|  | ANME-1b | 13.4% | 2 | ASV_35 (13.2%) |
|  | Anaerolineaceae | 7.6% | 29 | ASV_54 (6.2%) |
|  | Pirellulaceae | 7.0% | 24 | ASV_91 (4.6%) |
|  | Fermentibacteraceae | 5.6% | 23 | ASV_16 (1.8%), ASV_202 (1.1%) |
|  | ANME-1 | 4.4% | 12 | ASV_3 (4.2%) |
|  | ANME-2a-2b | 3.6% | 15 | ASV_133 (2.3%) |
| Elba Seep | ANME-1a | 44.7% | 1 | ASV_5 (44.7%) |
|  | Dissulfuribacteraceae | 10.1% | 11 | ASV_33 (6.3%), ASV_27 (3.3%) |
|  | AKAU3564 sediment group | 7.1% | 11 | ASV_38 (5.9%) |
|  | Desulfosarcinaceae | 5.2% | 11 | ASV_53 (4.2%) |
|  | Unclassified Bacteria | 5.1% | 7 | ASV_44 (5.0%) |
|  | ANME-2c | 4.4% | 1 | ASV_51 (4.4%) |
|  | ANME-2a-2b | 3.6% | 2 | ASV_66 (3.6%) |
| Guaymas Basin Methane 1G | Bathyarchaeia | 34.2% | 30 | ASV_8 (17.1%), ASV_9 (14.8%) |
|  | ANME-1 | 18.8% | 12 | ASV_3 (15.8%), ASV_47 (2.8%) |
|  | Aerophobales | 16.5% | 7 | ASV_13 (16.3%) |
|  | Desulfofervidaceae | 7.9% | 11 | ASV_30 (4.1%), ASV_2 (3.1%) |
|  | Thermodesulfobacteriaceae | 7.5% | 2 | ASV_19 (7.5%) |
|  | GN01 | 3.0% | 1 | ASV_56 (3.0%) |
| Guaymas Basin Methane G | Bathyarchaeia | 35.9% | 24 | ASV_8 (17.8%), ASV_9 (10.8%), ASV_55 (5.0%) |
|  | Desulfofervidaceae | 13.2% | 12 | ASV_2 (7.2%), ASV_30 (5.0%) |
|  | Aerophobales | 12.2% | 8 | ASV_26 (10.1%), ASV_13 (1.7%) |
|  | ANME-1 | 7.4% | 4 | ASV_3 (4.3%), ASV_47 (3.1%) |
|  | Thermodesulfobacteriaceae | 6.1% | 2 | ASV_19 (5.8%) |
|  | Aminicenantales | 5.0% | 4 | ASV_72 (2.8%), ASV_11 (2.1%) |
|  | Caldatribacteriaceae | 3.6% | 1 | ASV_76 (3.6%) |
| Guaymas Basin Methane 4 | ANME-1 | 53.8% | 16 | ASV_3 (52.1%) |
|  | Desulfofervidaceae | 29.0% | 13 | ASV_2 (28.2%) |
|  | Bathyarchaeia | 10.3% | 24 | ASV_9 (4.7%), ASV_8 (4.5%) |
| Guaymas Basin Hydrogen 1 | Archaeoglobaceae | 93.3% | 3 | ASV_1 (93.0%) |
|  | Desulfofervidaceae | 4.5% | 1 | ASV_2 (4.5%) |
| Guaymas Basin Hydrogen 3A | Archaeoglobaceae | 74.9% | 2 | ASV_1 (74.8%) |
|  | Desulfofervidaceae | 22.7% | 5 | ASV_2 (22.5%) |
| Guaymas Basin Propane | Desulfofervidaceae | 25.3% | 22 | ASV_2 (23.3%) |
|  | Syntrophoarchaeaceae | 13.6% | 6 | ASV_20 (13.3%) |
|  | JS1 | 8.7% | 4 | ASV_21 (8.6%) |
|  | Marine Benthic Group D and DHVEG-1 | 4.1% | 5 | ASV_102 (2.2%), ASV_144 (1.2%) |
| Guaymas Basin Butane | Aminicenantales | 23.4% | 4 | ASV_11 (23.4%) |
|  | Desulfofervidaceae | 9.9% | 9 | ASV_2 (9.6%) |
|  | Syntrophoarchaeaceae | 3.7% | 2 | ASV_107 (2.2%), ASV_20 (1.5%) |
|  | JS1 | 3.4% | 3 | ASV_21 (3.4%) |

**Table S2.** ASVs comprising at least 1% of total reads in the given sample, with their family-level classification. Only families >3% relative abundance are shown. The majority of families are comprised of no more than two ASVs.

|  | **Richness** | | **Simpson’s Evenness** | | **EQ** | | **Evar** | |
| --- | --- | --- | --- | --- | --- | --- | --- | --- |
| **Sample** | **Viral** | **Cellular** | **Viral** | **Cellular** | **Viral** | **Cellular** | **Viral** | **Cellular** |
| Santa Monica 3A | 313 | 259 | 0.00326246 | 0.05160458 | 0.06033558 | 0.10089224 | 0.0733037 | 0.18937416 |
| Santa Monica 3B | 395 | 230 | 0.00711382 | 0.05803197 | 0.05029099 | 0.0912637 | 0.04945798 | 0.15952748 |
| Santa Monica 3C | 384 | 209 | 0.01598118 | 0.01766819 | 0.04877084 | 0.09484208 | 0.04572982 | 0.18153026 |
| Santa Monica 4A | 412 | 260 | 0.0155338 | 0.0565383 | 0.0547177 | 0.0957181 | 0.05905509 | 0.18104189 |
| Santa Monica 4B | 422 | 363 | 0.01737859 | 0.04896974 | 0.0504078 | 0.1093796 | 0.04927744 | 0.23193287 |
| Santa Monica 4D | 417 | 342 | 0.02387932 | 0.08732644 | 0.05097117 | 0.11283316 | 0.05002626 | 0.23445262 |
| Guaymas Basin Hydrogen 1 | 333 | 40 | 0.03758998 | 0.0287957 | 0.06136846 | 0.07591399 | 0.07500451 | 0.11558463 |
| Guaymas Basin Hydrogen 3A | 319 | 66 | 0.03382399 | 0.02478285 | 0.06205792 | 0.08885116 | 0.07720127 | 0.20135376 |
| Guaymas Basin Methane 1G, 30% optiprep | 331 | N/A | 0.00413344 | N/A | 0.05902468 | N/A | 0.06943501 | N/A |
| Guaymas Basin Methane 1G, 35% optiprep | 325 | N/A | 0.00424544 | N/A | 0.05218896 | N/A | 0.0567324 | N/A |
| Guaymas Basin Methane 1G, 40% optiprep | 320 | N/A | 0.00935381 | N/A | 0.05221641 | N/A | 0.05509726 | N/A |
| Guaymas Basin Methane G | 330 | N/A | 0.00642214 | N/A | 0.05147287 | N/A | 0.05318532 | N/A |
| Guaymas Basin Methane 4 | 240 | 116 | 0.02751961 | 0.02244724 | 0.05047312 | 0.0951562 | 0.05046968 | 0.17643896 |
| Guaymas Basin Propane | 287 | 124 | 0.00395373 | 0.04127534 | 0.05846108 | 0.08940276 | 0.07136118 | 0.14667714 |
| Guaymas Basin Butane | 260 | 101 | 0.00915535 | 0.02717667 | 0.05765143 | 0.08865665 | 0.07108694 | 0.16991205 |
| Elba Seep | 376 | 202 | 0.02574961 | 0.02232657 | 0.05269071 | 0.09050086 | 0.05543185 | 0.16815102 |
| Menes Caldera 2 | 321 | 204 | 0.011038 | 0.04610654 | 0.04917194 | 0.09313789 | 0.049239 | 0.16557968 |
| Menes Caldera 2A |  | 197 |  | 0.08124868 |  | 0.09037035 |  | 0.16776429 |
| Black Sea | 377 | 460 | 0.040185 | 0.02340786 | 0.04503215 | 0.11170009 | 0.03978634 | 0.24550464 |

**Table S3.** Diversity metrics for host and viral communities.

|  | **Host 16S NMDS, ASV level** | **Host 16S NMDS, Species level** | **Viral PCA, Viral genus level** | **Viral PCA, Viral contig level** |
| --- | --- | --- | --- | --- |
| **Unique energy source** | 0.00039996 | 0.00089991 | 0.9290071 | 0.9527047 |
| **Methane vs non-methane energy source** | 0.00179982 | 0.00189981 | 0.3966603 | 0.4451555 |
| **Temperature lifestyle** | 9.999e-05 | 0.00019998 | 0.00019998 | 0.00029997 |
| **Original Sampling location** | 9.999e-05 | 9.999e-05 | 9.999e-05 | 9.999e-05 |

**Table S4.** PERMANOVA (ADONIS) values for ordination plots in Figures 2 and S1.

**Figure S1.** **A:** Viral CLR-transformed abundance-based PCA, at individual viral contig level. PC1 importance: 0.352, PC2 importance: 0.155 **B:** 16S relative abundance-based NMDS, at species taxonomy level. Stress=0.076.

**Figure S2.** Terminase large subunit tree, colored by sampling location. Ultrafast bootstrap values are indicated as colored dots on branch nodes; only values greater than 70% are shown.

**Figure S3.** **A)** Major Capsid Protein Tree, colored by incubation temperature. **B)** MCP tree colored by sampling location. Ultrafast bootstrap values are indicated as colored dots on branch nodes; only values greater than 70% are shown

**Figure S4.** **A)** Complete vConTACT2 network colored by incubation temperature. **B)** Complete vConTACT2 network colored by sampling location.

**Figure S5.** Heatmap showing average nucleotide identity between pairs of putative viral genomes. Clustering performed with Pheatmap (see methods), and each colorbar to the right of the heatmap indicates the temperature of the incubation from which the corresponding genome was derived. No clear pattern of clustering by incubation temperature is observed.

**Figure S6.** Full network of host-phage pairings. **A)** Network with phage genus-level clusters as nodes and shared host at the family level as edges. Nodes are labeled by host phylum; any viral cluster infecting *Desulfobacterota* is colored in shades of blue. **B)** Network with hosts at family taxonomic level as nodes and shared viral clusters as edges. Nodes are labeled by host phylum. Magenta nodes are families that are infected by broad host range viral cluster VC166, though VC166 may not be the only virus infecting that family. Node size corresponds to the number of viral clusters matched to each family via CRISPR spacers or tRNAs.

| **ORF** | **Vibrant Annotation** | **CD Search (e-value, bitscore)** | **GTDB r214 BLASTp top phyla (percent identity, e-value, bitscore)** | **NCBI BLASTp top phyla (percent identity, e-value, bitscore)** |
| --- | --- | --- | --- | --- |
| **45** | K02843 (*waaF*/*rfaF*) | *rfaF* superfamily (COG0859) (1.85e-16, 78.865) | *Desulfobacterota* (43.3, 7.23e-97, 301.0) | *Ca. Methanomethylicota* MAG (47.2, 4e-103, 318), *Thermodesulfobacteriota* MAG (43.31, 2e-97, 304) |
| **57** | K12547 (polysaccharidase *plyA*) | *nosD* (cl34609) with a choice-of-anchor Q domain (cl49486) (1.21e-10, 63.3972) | *KSB1* (39.2, 2.66e-118, 379.0), *Fermentibacterota* (41.7, 8.44e-112, 362.0), *OLB16* (39.5, 1.13e-111, 358.0) | *Bacteroidota* (41.03, 4e-137,428) |
| **74** | K03269 (*lpxH*) | Metallophosphatase superfamily (cl13995 and cl42652) (2.36e-12, 63.5309) | *Thermoplasmatota* (33.2, 1.01e-22, 101.0), *UBP14* (30.8, 1.88e-21, 98.6) | *Bathyarchaeota* MAG (62.26, 3e-93, 283), *Thorarchaeota* MAG (62.26, 1e-92, 182) |
| **82** | K01448 (*amiABC*) | *amiC* family (COG0860) (5.77e-71, 213.204) | *Methanobacteriota* (45.4, 2.43e-37, 140.0, *Bacillota* (45.4, 3.42e-37, 140.0) | *Thermodesulfobacteriota* (*D. auxilii*) MAG (44.89, 5e-38, 140), *Methanobacteriota* (45.65, 1e-37, 143) |
| **112** | Hypothetical protein | Uncharacterized conserved protein w/ vonWillibrand factor type A (cl27002) (9.72e-05, 44.2856) | *Spirochaetota* (e=2.930000e-115, bitscore=360.0) | *Synergistota* MAG (45.66, 2e-140, 426), *Methanobacteriota* MAG (44.06, 2e-139, 423) |
| **109** | K03148 (*thiF*) | E1 activating enzymes of ubiquitin-like proteins (cl22428) (1.84e-15, 71.0409) | *Myxococcota* (34.3, 1.01e-13, 76.3) | *Methanobacteriota* MAG (37.82, 9e-22, 99.0) |
| **16** | K07391 (*comM*) | *YifB* superfamily (cl33973) (5.67e-08, 51.1944) | *Bacillota* (29.0, 3.0e-6, 57.4) | *Deltaproteobacteria* MAG (31.21, 5e-16, 83.2) |
| **84** | K00558 (DNMT1) | *Dcm* DNA-cytosine methylase (COG0270) (7.68e-66, 206.198) | *Halobacteriota* (42.3, 8.71e-50, 173.0) | *Nitrospirota* MAG (41.84, 2e-54, 187) |

**Table S5.** BLAST and conserved domain search results at the phylum level for open reading frames from contig f9bf2df06235b506f7396f56f61038ed in VC143. Though the *plyA* annotation was found to have some conserved domain similarities to *nosD*, it was not found to be a high quality *nosD*-like sequence in a search with the *nosD* HMM.

**Figure S7.** Complete list of AMGs and their abundance in each sample. See main text Figure 6 for information on abundance calculations.

**Figure S8.** Examples of *nosD*-like proteins spanning the conserved His-Met-Met motif. Sequences labeled as *Reference nosD* are canonical *nosD* sequences.

**Figure S9.** Structures of *nosD*-like proteins from this study as predicted by Alphafold2, labeled by the fasta header of the viral contig from which they came. The *nosD*-like protein is colored by confidence values (pLDDT, as calculated by Alphafold) and is superimposed on the structure of the canonical *nosD* in grey. A few sample structures were predicted with both Alphafold2 and Alphafold3, with the latter prediction marked with an asterisk (*).

## Supplementary Methods

*Development and maintenance of incubations*

Anaerobic alkane-oxidizing enrichments from Guaymas Basin, Elba, Menes Caldera, and the Black Sea were developed via dilution of sediment slurries with anoxic artificial seawater medium: 150mL slurries (150 ml) were transferred in 256 ml serum bottles sealed with butyl rubber stoppers [7]. Inoculum for the Santa Monica Basin enrichment cultures was created using Percoll density separation and filtration under anoxic conditions to concentrate methane-oxidizing consortia away from sediment particles [1]. All incubations are maintained in an artificial seawater medium containing sulfate as the sole electron acceptor. Sulfide concentrations were measured to track metabolic activity, and active cultures were consecutively diluted. Spent media from 100mL incubations was replaced by allowing the particles to settle and removing 80mL of medium and replacing the same volume with new anoxic artificial seawater. The *D. auxilii* and *Archaeoglobus* incubations were maintained under 0.2 MPa of 80:20 hydrogen:carbon dioxide, while the AOM enrichments were provided 0.2MPa of 100% methane. Propane and butane enrichments were produced as described above from Guaymas Basin, and slurries and their dilutions were kept at 0.15 MPa propane/butane. A full description is provided in Supplementary Table S1, including references for media composition.

*Media recipe for Guaymas Basin Methane and Hydrogen incubations*

1. Begin with the following in 800mL of water, for a final volume of 1L:

| **Reagent** | **g or mL/L** | **g/mol** | **final mM concentration** |
| --- | --- | --- | --- |
| MgCl_2_・6H_2_O | 5.67g | 203.3 | 27.9 |
| CaCl_2_・2H_2_O | 0.22g | 147.0 | 1.5 |
| NaCl | 26.37g | 58.4 | 451.2 |
| KCl | 0.60g | 74.6 | 8.0 |
| Na_2_SO_4_ | 1.50g | 142.0 | 10.6 |
| K_2_HPO_4_ | 0.17g | 174.2 | 1.0 |
| NH_4_Cl | 0.11g | 53.5 | 2.0 |
| Se/W solution (see below) | 0.10mL |  | 0.01/0.0067 uM |
| Trace elements solution (see below) | 1.00mL |  |  |
| 250mM HEPES buffer (see below) | 100.00mL |  | 25.0 |

2. The pH should be between 7.2-7.45. Filter through a 0.22µm pore size filter to sterilize and flush with nitrogen gas for 20-30 minutes to make the media anoxic.
3. Add the following after the media has been flushed:

| **Reagent** | **g or mL/L** | **final mM concentration** |
| --- | --- | --- |
| 200mM HS^-^ solution, flushed with nitrogen gas | 2.5mL | 0.5 |
| 1M NaHCO_3_^-^ solution, filter sterilized and flushed with nitrogen gas | 5mL | 5 |

Se/W solution and trace element solution recipes can be found in previously published work [8]. For a 500mL stock solution of 250mM HEPES at pH 7.5, use 32.5g HEPES and 2.7g of NaOH.

*Collection and purification of viruses*

Spent media from the enrichment incubations were removed and filtered through a 0.22µm pore size polyethersulfone membrane (Cat. SLGP033N, Millipore Sigma; St. Louis, MO, USA) to remove cells and cell debris. Viruses in the filtrate were concentrated in an Amicon Ultra-15 Centrifugal Filter Unit with a 100kDa cutoff (Cat. UFC910024, Millipore Sigma; St. Louis, MO, USA) spun at 2000×g for 3 minutes (Allegra X-I5R Centrifuge, Beckman Coulter; Brea, CA, USA). To increase yield of viruses from the filtration membrane, the cartridge was wrapped with parafilm and vortexed at the maximum setting for 10 seconds (Vortex Genie 2, Scientific Industries; Bohemia, NY, USA). Concentrated viruses were then purified using ultracentrifugation (Ultima MAX-E Ultracentrifuge; Beckman Coulter, Brea, CA, USA) with Optiprep Density Gradient Medium (Cat. D1556, Sigma Aldrich; St. Louis, MO, USA) using a protocol adapted from previously published work [9] and detailed in publicly available documentation [10]. Density fractions were then stained with SYBR™ Gold Nucleic Acid Gel Stain (Cat. S11494, ThermoFisher; Waltham, MA, USA) [11] and visualized with fluorescence microscopy to determine which fractions contained viral particles. The fractions marked by 30%, 35%, and 40% were found to contain the largest number of viral particles. Note that this method does not account for the presence of non-viral particles containing nucleic acid, such as vesicles, but vesicles are thought to affect total viral counts by less than an order of magnitude in seawater samples [12].

*Transmission Electron Microscopy*

Spent media from incubations was collected, filtered, and concentrated as described under *Collection and purification of viruses*. Five microliters of virus concentrate were spotted on a glow-discharged carbon-coated 300 mesh copper TEM grid (Cat. 01813, Ted Pella Inc., Redding, CA, USA). Samples were incubated on the grid at room temperature for five minutes, and excess liquid was wicked off with filter paper. Once dry, grids were stained with 2% uranyl acetate solution in 1X PBS for 30 seconds; the staining procedure was repeated twice for a total of 60 seconds of treatment. Excess stain was removed and grids were air dried completely before imaging with an FEI Tecnai T12 at 120kV. Electron microscopy was performed in the Beckman Institute Resource Center for Transmission Electron Microscopy at Caltech.

*Viral DNA extraction and sequencing*

Density fractions were treated with RNase-free DNaseI (New England Biolabs M0303L) to remove free DNA. Viral DNA was extracted using Promega Wizard PCR Preps DNA Purification Resin (Cat. A7181, Promega, Madison, WI, USA) and Wizard Minicolumns (Cat. A7211, Promega, Madison, WI, USA) per published protocols [13]. Sequencing libraries were prepared using the Illumina DNA Prep library kit (Cat 20018705, Illumina, San Diego, CA, USA) with IDT for Illumina DNA/RNA UD Indexes Set C (Cat. 20042666). The DNA from the two Menes Caldera incubations was pooled to ensure sufficient input for library preparation. Paired-end sequencing was performed at the University of Southern California Keck School of Medicine using an S1 flow cell for 300 cycles (150bp reads) on the NovaSeq6000 System using v1.5 reagents (Illumina).

*Cellular DNA extraction and sequencing*

Approximately 15mL of each enrichment was collected and centrifuged at 5000×g and 10˚C for 30 minutes. The supernatant was removed and the resulting cell pellet was extracted using Qiagen’s DNeasy Blood and Tissue Kit according to their Gram positive protocol (Cat. #69504, Qiagen, Hilden, Germany). 16S rRNA archaeal and bacterial genes were amplified in duplicate with Q5 Hot Start Master Mix (Cat. M0492S, New England Biolabs, Ipswich, MA, USA) according to the manufacturer’s directions, using 515F (5’-TCGTCGGCAGCGTCAGATGTGTATAAGAGACAG-GTGYCAGCMGCCGCGGTAA-3’) and 926R (5’-GTCTCGTGGGCTCGGAGATGTGTATAAGAGACAG-CCGYCAATTYMTTTRAGTTT-3’) primers [14] that include Illumina adapters for 32 PCR cycles. PCR duplicates were then pooled and barcoded with the Illumina Nextera XT Index Kit v2. Amplification was performed with Q5 Hot Start PCR mixture and 3μL of the pooled duplicate product was added to a 30μL reaction volume, annealed at 66°C, and cycled 11 times. Products were run on a 1.5% agarose gel and quantified by band intensity. These barcoded PCR products were then combined in equimolar amounts into a single tube and 300uL of this pooled sample was run on a 1.5% low melt agarose gel (Cat. BP165-25, ThermoFisher) and purified using Promega’s Wizard SV Gel and PCR Clean-up System (Cat. #A928, Promega, Madison, WI, USA). The sample was sequenced by Laragen (Culver City, CA, USA) using the MiSeq Reagent Kit v3 (Cat. #MS-102-3003, 600-cycle) on the MiSeq System (Illumina) with the addition of 15-20% PhiX. Figure 1 was generated after dropping singletons and organisms classified as “uncultured” at the family level. The 16S rRNA gene sequencing data was not included for Guaymas Basin Methane 1G and Guaymas Basin Methane G due to a significant temporal gap between virus sampling and 16S rRNA gene sampling, in which the bottles were diluted into larger containers. 16S rRNA amplicon data was processed with the DADA2 v.1.22 workflow [15]. A reproducible workflow is available in the Caltech Library data repository (see Data Availability statement). SILVA SSU database r138 [16] with in-house sequences added was used to assign taxonomy to amplicon sequence variants. Whole genome library preparation and paired-ended sequencing was performed at the Millard and Muriel Jacobs Genetics and Genomics Laboratory, California Institute of Technology, using a P1 flowcell for 300 cycles (150bp reads) with the NextSeq2000 System (Illumina).

*Cellular metagenome assembly and binning*

Reads were trimmed with bbduk v38.81 [17] and filtered such that reads with <150 nucleotides were eliminated. Reads that passed this step were assembled using metaSPAdes version 3.15.3 [18]. Reads were aligned to the assembled contigs using the Burrows-Wheeler Alignment tool version 0.7.17-r1188 [19] before contigs were binned using MetaBAT version 2.12.1 [20] with a minimum contig length of 1500 bp. The CheckM v1.1.3 lineage workflow [21] was used to determine the quality of each bin. Taxonomy was assigned with GTDB-Tk v2.4.1 [22] using the GTDB R226 database [23–26]. All reads were also mapped back to the full set of contigs using bbmap v38.81 [17]; unmapped reads were reassembled with metaSPAdes.

*vMAG assembly, annotation, and phylogeny*

Viral reads were trimmed using bbduk v38.81 with kmer lengths of 23, minimum kmer length of 11, maximum hamming distance of 1, and parameters set to trim evenly at the overlap of paired reads. Contigs were assembled using metaViralSPAdes v3.15.3 [27] with default parameters. To improve the quality of assembly and number of complete vMAGs, all Guaymas Basin methane incubation reads were pooled before assembly, as were Guaymas Bain hydrogen reads and Santa Monica reads. Contigs were classified as viral based on identification using ViralVerify [27], Seeker [28], or Vibrant [29], and if more than 50% of the predicted genes were categorized as viral rather than cellular by checkV [30]. viralVerify was also set to distinguish plasmids. Comparison to the IMGVR v4 database [31] and Costa Rica metaviromes [32] was performed using MUMmer’s nucmer v3.1 program [33], with the mincluster set to 100. All virome-assembled contigs, regardless of whether they were identified as viral by the preceding algorithms, were subject to annotation by Vibrant [29]; Vibrant filters based on probable viral identity before annotation, adding a fourth set of checks to the three algorithms cited above. All cellular contigs (see *Cellular Metagenome Assembly and Binning*) were checked to identify viruses in the cellular metagenomes; these viral contigs were also annotated with Vibrant and combined with the vMAGs from the viral sequencing for downstream work.

Terminase large subunit (*TerL*) and major capsid protein (*MCP*) phylogenies were constructed using the annotations obtained from Vibrant. All genes were single copy. Relevant amino acid sequences were aligned using MAFFT v7.505 [34] and trimmed with trimAl v1.4.rev15 [35]. Alignments were manually curated in Jalview v1.8 [36] by calculating a tree using neighbor joining methods, then removing short sequences (<200 amino acids) or unclustered sequences (i.e. those not part of an obvious cluster in the tree) with multiple large gaps (>30 amino acids). Phylogenetic trees were generated using IQ-TREE multicore version 1.6.12 [37].

Auxiliary metabolic genes were included in the analysis if classified as such by Vibrant. Because annotations and open reading frame calls are not always reliable without a great deal of manual curation, AMG abundances per sample were quantified as the abundance of the viral contig on which the AMG was found (see *Viral Abundance Calculations and Diversity*) rather than the abundance of the AMG itself. Within a given sample, a contig was included in the AMG calculations if the centered log ratio of the contig’s abundance was >1.

*Viral classification and clustering*

Viral contigs that were 98% complete or higher according to CheckV were classified at the family level using GRAViTy: Genome Relationships Applied to Virus Taxonomy [38], with ICTV Virus Metadata Resource VMR_16-180521_MSL36 (<https://ictv.global/vmr>, [39]). The whole-genome protein clustering network was generated using vConTACT2 [40] using Refseq release 207, pcs mode MCP, vcs mod ClusterONE. The network was visualized using Cytoscape. ANME-1 phages from a previous study in Southern Pescadero Basin [41] were included in the network calculation due to the similarities of the sampling site and host taxonomy to those used in this study. T7 control sequences were included in vConTACT2 analysis as an internal check.

Average nucleotide identity was calculated using the script anicalc.py and clusters were found using the script aniclust.py from the checkV repository (accessed October 20, 2025) [30]. Visualization was performed using the R package pheatmap using the centroid clustering option [42].

*Viral abundance calculations and ordination*

Viral reads were quantified per viral cluster and per contig using salmon v1.10.0 [43] and summarized using tximport v1.18.0 [44]. DESeq2 v1.30.1 [45] was used to quantify the abundance of each cluster/contig per library. Abundances were centered log ratio (CLR) transformed with ALDEx2 v1.25.1 [46]. Diversity metrics were calculated using the transcripts per million value for each viral cluster, as output by tximport. Principal components, corresponding PERMANOVA (Adonis, analysis of dissimilarities) values, and rank abundances were calculated using the CLR-transformed data.

Host community nonmetric multidimensional scaling (NMDS) ordination plots were generated using the vegan v2.6-4 [47] metaMDS function (Bray-Curtis dissimilarity metric on relative abundance data) at the amplicon sequence variant (ASV) level [48]. Viral community principal component analysis (PCA) ordination plots were generated using the prcomp function (Euclidean distance on centered log ratio transformed abundance data) at the viral genus level; principal component analysis (PCA) was used for the viral community instead of NMDS because the centered log ratio transformation moves compositional data into Euclidean space [49]. For both host and virus communities, corresponding PERMANOVA values were calculated with the vegan adonis2 function.

*CRISPR and tRNA identification and host matching*

Potential cellular host genomes were obtained from NCBI from Projects PRJNA276404 [2, 50]; PRJNA318983 & PRJNA319143 [4]; PRJNA418316 [51]; PRJEB36446 & PRJEB36096 [52]; PRJNA713414 [53]; PRJNA758896 [1]; PRJNA875076 & PRJNA721962 [41]; members of *Ca. Desulfofervidaceae* from PRJNA762493 [54]; and personal communications with Dr. Daan Speth at the University of Vienna and Dr. Rafael Laso-Pérez at the Museo Nacional de Ciencias Naturales, Madrid. These genomes were generated from bottles used in this study or were sampled from closely-related sites. Sixty-three additional *Archaeoglobus* genomes were obtained from GTDB under the search time “archaeoglobus”. Additional cellular genomes were generated from three of the incubation bottles (Guaymas Basin Methane 1G, Guaymas Basin Methane G, and Guaymas Basin Hydrogen 3A) as detailed above.

CRISPRs were called using CRISPRDetect 3.0 [55] and CRISPRCasTyper v1.1.4 [56]. Host genome CRISPR spacers were combined and dereplicated with MMseqs2 [57] version 13.45111easy-cluster workflow at 99% minimum sequence identity and 80% coverage. BLASTn v2.12.0+ and SpacePharer v5.c2e680a [58] were used to compare the viral contigs and the host spacers. BLAST hits were considered high-quality with at least 98% sequence similarity and no more than 2 mismatches.

tRNAs were identified in putative host genomes and viral genomes using tRNAscan-SE v2.0.5 [59] and Aragorn v1.2.41 [60]. Viral and host tRNAs were combined and dereplicated respectively, as done for the CRISPRs. Host tRNAs were aligned against viral contigs and viral tRNAs using BLAST. Matches were considered high-quality only if they were exact matches: 100% sequence identity, 100% coverage, and no mismatches.

Host/phage network diagrams were made using adjacency matrices of viral clusters that shared hosts, and hosts infected by the same viral clusters, respectively. Where viruses were not clustered at the genus level by vConTACT2, the contig was used on its own. Networks were calculated using the networkX from_pandas_adjacency function [61], and self-loops were removed. The network was visualized using Cytoscape [62]. The genome map of VC143 was visualized using DNA Features Viewer [63].

iPHoP-based classification [64] was performed using the default settings and the June 2025 iPHoP database. Matches were filtered for confidence scores >95.

*Identification of nosD-like proteins using a hidden Markov model*

Input sequences for the *nosD* Hidden Markov Model were derived from Extended Data Figure 7A of a previous study concerning nosD structure [65]. Sequences for *Geobacillus thernodenitrificans* (WP_008880109.1), *Shewanella denitrificans* (WP_011496651.1), *Stutzerimonas stutzeri* (WP_036990777.1), *Marinobacter nauticus* (WP_342631055.1), *Wolinella succinogens* (WP_011138819.1), *Hydrogenobacter thermophilus* (WP_012962810.1), and *Deslfitobacterium* hafniense (WP_018214548.1) were aligned against a curated set of genomes (See Caltech Library link under Data Availability) using BLAST v2.9.0+. Hits with e-value<0.0005 and bitscore>200 were retained and aligned with the selected sequences from the prior structural study of *nosD* [65] using MUSCLE v3.8.31 [66]. The alignment was manually curated and dereplicated at 70% identity in Jalview [36].

The HMM was built from this alignment; all viral proteins output by Vibrant were searched for *nosD*-like proteins [67]. *nosD*-like proteins were included in this study if they had an hmmsearch e-value<0.001, came from contigs that were confirmed to be viruses using the criteria under *vMAG Assembly and Annotation*, came from contigs at least 5000 basepairs in length, and had a greater proportion of viral genes than host genes according to checkV. Vibrant *nosD* annotations that were not found in the hmmsearch results were included in the study if they were >200 amino acids in length and appeared to span the conserved His-Met-Met residues of the canonical *nosD* proteins [65] in a MUSCLE-generated alignment. These open reading frames were truncated to remove any additional protein domains before inclusion in the phylogeny; the presence of additional domains and the location within the ORF of the nosD-like protein were determined using NCBI’s Conserved Domain Search.

Viral *nosD*-like proteins from the Tara Oceans and Malaspina viral study [68], Costa Rica methane cold seeps [32, 69], lake sediment and water column [70], and soils [71] were similarly searched using the *nosD* HMM and thresholded at e-value<0.001. For published datasets that were only available as nucleic acid fasta files, genes were predicted with Prodigal V2.6.3: February, 2016 [72] before hmmsearch was run. Alignments were generated with MUSCLE v3.8.31 [66] and trees were inferred using IQ-TREE2 [37] using the model finder mode and confidence in tree topology was evaluated with an ultrafast bootstrap value of 1000. Protein structures were predicted using Alphafold2 [73] and/or Alphafold3 [74] in monomer mode with the reduced set of databases (MGnify, PDB70 (with PDBx/mmCIF and PDB SEQRES), BFD, Uniclust30, Uniprot, and Uniref90) and visualized using ChimeraX [75–77].

**Supplementary References**

1.	Yu H, Speth DR, Connon SA, et al. Community Structure and Microbial Associations in Sediment-Free Methanotrophic Enrichment Cultures from a Marine Methane Seep. *Appl Environ Microbiol* 2022;e02109-21. https://doi.org/10.1128/aem.02109-21

2.	Krukenberg V, Harding K, Richter M, et al. Candidatus Desulfofervidus auxilii, a hydrogenotrophic sulfate-reducing bacterium involved in the thermophilic anaerobic oxidation of methane: The partner bacterium in thermophilic methanotrophy. *Environ Microbiol* 2016;**18**:3073–3091. https://doi.org/10.1111/1462-2920.13283

3.	Holler T, Widdel F, Knittel K, et al. Thermophilic anaerobic oxidation of methane by marine microbial consortia. *ISME J* 2011;**5**:1946–1956. https://doi.org/10.1038/ismej.2011.77

4.	Laso-Pérez R, Wegener G, Knittel K, et al. Thermophilic archaea activate butane via alkyl-coenzyme M formation. *Nature* 2016;**539**:396–401. https://doi.org/10.1038/nature20152

5.	Ruff SE, Kuhfuss H, Wegener G, et al. Methane Seep in Shallow-Water Permeable Sediment Harbors High Diversity of Anaerobic Methanotrophic Communities, Elba, Italy. *Front Microbiol* 2016;**7**. https://doi.org/10.3389/fmicb.2016.00374

6.	Wegener G, Krukenberg V, Ruff SE, et al. Metabolic Capabilities of Microorganisms Involved in and Associated with the Anaerobic Oxidation of Methane. *Front Microbiol* 2016;**7**. https://doi.org/10.3389/fmicb.2016.00046

7.	Laso-Pérez R, Krukenberg V, Musat F, et al. Establishing anaerobic hydrocarbon-degrading enrichment cultures of microorganisms under strictly anoxic conditions. *Nat Protoc* 2018;**13**:1310–1330. https://doi.org/10.1038/nprot.2018.030

8.	Scheller S, Yu H, Chadwick GL, et al. Artificial electron acceptors decouple archaeal methane oxidation from sulfate reduction. *Science* 2016;**351**:703–707. https://doi.org/10.1126/science.aad7154

9.	Kauffman KM, Hussain FA, Yang J, et al. A major lineage of non-tailed dsDNA viruses as unrecognized killers of marine bacteria. *Nature* 2018;**554**:118–122. https://doi.org/10.1038/nature25474

10.	Narayanan AK, Philosof A. Opti-Prep density separation of viruses for metagenomics v1. 2020. https://doi.org/10.17504/protocols.io.261geobqwl47/v1

11.	Narayanan AK, Philosof A. SYBR Gold staining of viral-like particles on Anodiscs v1. 2024. https://doi.org/10.17504/protocols.io.8epv5rd85g1b/v1

12.	Biller SJ, McDaniel LD, Breitbart M, et al. Membrane vesicles in sea water: heterogeneous DNA content and implications for viral abundance estimates. *ISME J* 2017;**11**:394–404. https://doi.org/10.1038/ismej.2016.134

13.	Poulos B. DNA Extraction of Viruses Using Wizard Columns Version 1. 2012. Tucson Marine Phage Lab, 2012.

14.	Parada AE, Needham DM, Fuhrman JA. Every base matters: assessing small subunit rRNA primers for marine microbiomes with mock communities, time series and global field samples. *Environ Microbiol* 2016;**18**:1403–1414. https://doi.org/10.1111/1462-2920.13023

15.	Callahan BJ, McMurdie PJ, Rosen MJ, et al. DADA2: High-resolution sample inference from Illumina amplicon data. *Nat Methods* 2016;**13**:581–583. https://doi.org/10.1038/nmeth.3869

16.	Quast C, Pruesse E, Yilmaz P, et al. The SILVA ribosomal RNA gene database project: improved data processing and web-based tools. *Nucleic Acids Res* 2012;**41**:D590–D596. https://doi.org/10.1093/nar/gks1219

17.	Bushnell, Brian. BBTools.

18.	Nurk S, Meleshko D, Korobeynikov A, et al. metaSPAdes: a new versatile metagenomic assembler. *Genome Res* 2017;**27**:824–834. https://doi.org/10.1101/gr.213959.116

19.	Li H, Durbin R. Fast and accurate short read alignment with Burrows-Wheeler transform. *Bioinforma Oxf Engl* 2009;**25**:1754–1760. https://doi.org/10.1093/bioinformatics/btp324

20.	Kang DD, Froula J, Egan R, et al. MetaBAT, an efficient tool for accurately reconstructing single genomes from complex microbial communities. *PeerJ* 2015;**3**:e1165. https://doi.org/10.7717/peerj.1165

21.	Parks DH, Imelfort M, Skennerton CT, et al. CheckM: assessing the quality of microbial genomes recovered from isolates, single cells, and metagenomes. *Genome Res* 2015;**25**:1043–1055. https://doi.org/10.1101/gr.186072.114

22.	Chaumeil P-A, Mussig AJ, Hugenholtz P, et al. GTDB-Tk v2: memory friendly classification with the genome taxonomy database. *Bioinformatics* 2022;**38**:5315–5316. https://doi.org/10.1093/bioinformatics/btac672

23.	Parks DH, Chuvochina M, Waite DW, et al. A standardized bacterial taxonomy based on genome phylogeny substantially revises the tree of life. *Nat Biotechnol* 2018;**36**:996–1004. https://doi.org/10.1038/nbt.4229

24.	Parks DH, Chuvochina M, Chaumeil P-A, et al. A complete domain-to-species taxonomy for Bacteria and Archaea. *Nat Biotechnol* 2020;**38**:1079–1086. https://doi.org/10.1038/s41587-020-0501-8

25.	Parks DH, Chuvochina M, Rinke C, et al. GTDB: an ongoing census of bacterial and archaeal diversity through a phylogenetically consistent, rank normalized and complete genome-based taxonomy. *Nucleic Acids Res* 2022;**50**:D785–D794. https://doi.org/10.1093/nar/gkab776

26.	Rinke C, Chuvochina M, Mussig AJ, et al. A standardized archaeal taxonomy for the Genome Taxonomy Database. *Nat Microbiol* 2021;**6**:946–959. https://doi.org/10.1038/s41564-021-00918-8

27.	Antipov D, Raiko M, Lapidus A, et al. MetaviralSPAdes: assembly of viruses from metagenomic data. *Bioinformatics* 2020;**36**:4126–4129. https://doi.org/10.1093/bioinformatics/btaa490

28.	Auslander N, Gussow AB, Benler S, et al. Seeker: alignment-free identification of bacteriophage genomes by deep learning. *Nucleic Acids Res* 2020;**48**:e121–e121. https://doi.org/10.1093/nar/gkaa856

29.	Kieft K, Zhou Z, Anantharaman K. VIBRANT: automated recovery, annotation and curation of microbial viruses, and evaluation of viral community function from genomic sequences. *Microbiome* 2020;**8**:90. https://doi.org/10.1186/s40168-020-00867-0

30.	Nayfach S, Camargo AP, Schulz F, et al. CheckV assesses the quality and completeness of metagenome-assembled viral genomes. *Nat Biotechnol* 2021;**39**:578–585. https://doi.org/10.1038/s41587-020-00774-7

31.	Camargo AP, Nayfach S, Chen I-MA, et al. IMG/VR v4: an expanded database of uncultivated virus genomes within a framework of extensive functional, taxonomic, and ecological metadata. *Nucleic Acids Res* 2023;**51**:D733–D743. https://doi.org/10.1093/nar/gkac1037

32.	Philosof A, Narayanan AK, Murali R, et al. Phage infection networks spanning distant taxonomic groups are a defining feature of deep marine methane seeps. *Prep* .

33.	Kurtz S, Phillippy A, Delcher AL, et al. Versatile and open software for comparing large genomes. *Genome Biol* 2004;**5**:R12. https://doi.org/10.1186/gb-2004-5-2-r12

34.	Katoh K, Standley DM. MAFFT Multiple Sequence Alignment Software Version 7: Improvements in Performance and Usability. *Mol Biol Evol* 2013;**30**:772–780. https://doi.org/10.1093/molbev/mst010

35.	Capella-Gutiérrez S, Silla-Martínez JM, Gabaldón T. trimAl: a tool for automated alignment trimming in large-scale phylogenetic analyses. *Bioinformatics* 2009;**25**:1972–1973. https://doi.org/10.1093/bioinformatics/btp348

36.	Waterhouse AM, Procter JB, Martin DMA, et al. Jalview Version 2—a multiple sequence alignment editor and analysis workbench. *Bioinformatics* 2009;**25**:1189–1191. https://doi.org/10.1093/bioinformatics/btp033

37.	Nguyen L-T, Schmidt HA, Von Haeseler A, et al. IQ-TREE: A Fast and Effective Stochastic Algorithm for Estimating Maximum-Likelihood Phylogenies. *Mol Biol Evol* 2015;**32**:268–274. https://doi.org/10.1093/molbev/msu300

38.	Aiewsakun P, Adriaenssens EM, Lavigne R, et al. Evaluation of the genomic diversity of viruses infecting bacteria, archaea and eukaryotes using a common bioinformatic platform: steps towards a unified taxonomy. *J Gen Virol* 2018;**99**:1331–1343. https://doi.org/10.1099/jgv.0.001110

39.	Lefkowitz EJ, Dempsey DM, Hendrickson RC, et al. Virus taxonomy: the database of the International Committee on Taxonomy of Viruses (ICTV). *Nucleic Acids Res* 2018;**46**:D708–D717. https://doi.org/10.1093/nar/gkx932

40.	Bolduc B, Jang HB, Doulcier G, et al. vConTACT: an iVirus tool to classify double-stranded DNA viruses that infect *Archaea* and *Bacteria*. *PeerJ* 2017;**5**:e3243. https://doi.org/10.7717/peerj.3243

41.	Laso-Pérez R, Wu F, Crémière A, et al. Evolutionary diversification of methanotrophic ANME-1 archaea and their expansive virome. *Nat Microbiol* 2023. https://doi.org/10.1038/s41564-022-01297-4

42.	Kolde R. pheatmap. 2025. 2025.

43.	Patro R, Duggal G, Love MI, et al. Salmon provides fast and bias-aware quantification of transcript expression. *Nat Methods* 2017;**14**:417–419. https://doi.org/10.1038/nmeth.4197

44.	Soneson C, Love MI, Robinson MD. Differential analyses for RNA-seq: transcript-level estimates improve gene-level inferences. *F1000Research* 2015;**4**:1521. https://doi.org/10.12688/f1000research.7563.1

45.	Love MI, Huber W, Anders S. Moderated estimation of fold change and dispersion for RNA-seq data with DESeq2. *Genome Biol* 2014;**15**:550. https://doi.org/10.1186/s13059-014-0550-8

46.	Fernandes AD, Macklaim JM, Linn TG, et al. ANOVA-Like Differential Expression (ALDEx) Analysis for Mixed Population RNA-Seq. *PLoS ONE* 2013;**8**:e67019. https://doi.org/10.1371/journal.pone.0067019

47.	Oksanen J, Simpson GL, Blanchet FG, et al. vegan: Community Ecology Package.

48.	McArdle BH, Anderson MJ. FITTING MULTIVARIATE MODELS TO COMMUNITY DATA: A COMMENT ON DISTANCE-BASED REDUNDANCY ANALYSIS. *Ecology* 2001;**82**:290–297. https://doi.org/10.1890/0012-9658(2001)082%5B0290:FMMTCD%5D2.0.CO;2

49.	Quinn TP, Erb I, Richardson MF, et al. Understanding sequencing data as compositions: an outlook and review. *Bioinformatics* 2018;**34**:2870–2878. https://doi.org/10.1093/bioinformatics/bty175

50.	Wegener G, Krukenberg V, Riedel D, et al. Intercellular wiring enables electron transfer between methanotrophic archaea and bacteria. *Nature* 2015;**526**:587–590. https://doi.org/10.1038/nature15733

51.	Krukenberg V, Riedel D, Gruber-Vodicka HR, et al. Gene expression and ultrastructure of meso- and thermophilic methanotrophic consortia: Interspecies interaction within AOM consortia. *Environ Microbiol* 2018;**20**:1651–1666. https://doi.org/10.1111/1462-2920.14077

52.	Hahn CJ, Laso-Pérez R, Vulcano F, et al. “Candidatus Ethanoperedens,” a Thermophilic Genus of Archaea Mediating the Anaerobic Oxidation of Ethane. 2020;**11**:18.

53.	Speth DR, Yu FB, Connon SA, et al. Microbial communities of Auka hydrothermal sediments shed light on vent biogeography and the evolutionary history of thermophily. *ISME J* 2022;**16**:1750–1764. https://doi.org/10.1038/s41396-022-01222-x

54.	Murali R, Yu H, Speth DR, et al. Physiological potential and evolutionary trajectories of syntrophic sulfate-reducing bacterial partners of anaerobic methanotrophic archaea. *PLOS Biol* 2023;**21**:e3002292. https://doi.org/10.1371/journal.pbio.3002292

55.	Biswas A, Staals RHJ, Morales SE, et al. CRISPRDetect: A flexible algorithm to define CRISPR arrays. *BMC Genomics* 2016;**17**:356. https://doi.org/10.1186/s12864-016-2627-0

56.	Russel J, Pinilla-Redondo R, Mayo-Muñoz D, et al. CRISPRCasTyper: Automated Identification, Annotation, and Classification of CRISPR-Cas Loci. *CRISPR J* 2020;**3**:462–469. https://doi.org/10.1089/crispr.2020.0059

57.	Steinegger M, Söding J. MMseqs2 enables sensitive protein sequence searching for the analysis of massive data sets. *Nat Biotechnol* 2017;**35**:1026–1028. https://doi.org/10.1038/nbt.3988

58.	Zhang R, Mirdita M, Levy Karin E, et al. SpacePHARER: sensitive identification of phages from CRISPR spacers in prokaryotic hosts. *Bioinformatics* 2021;**37**:3364–3366. https://doi.org/10.1093/bioinformatics/btab222

59.	Chan PP, Lin BY, Mak AJ, et al. tRNAscan-SE 2.0: improved detection and functional classification of transfer RNA genes. *Nucleic Acids Res* 2021;**49**:9077–9096. https://doi.org/10.1093/nar/gkab688

60.	Laslett D. ARAGORN, a program to detect tRNA genes and tmRNA genes in nucleotide sequences. *Nucleic Acids Res* 2004;**32**:11–16. https://doi.org/10.1093/nar/gkh152

61.	Hagberg AA, Schult DA, Swart PJ. “Exploring network structure, dynamics, and function using NetworkX”. 2008. Pasadena, CA: Proceedings of the 7th Python in Science Conference, 2008, pp 11–15.

62.	Shannon P, Markiel A, Ozier O, et al. Cytoscape: A Software Environment for Integrated Models of Biomolecular Interaction Networks. *Genome Res* 2003;**13**:2498–2504. https://doi.org/10.1101/gr.1239303

63.	Zulko. DNA Features Viewer. 2016. Edinburgh Genome Foundry, 2016.

64.	Roux S, Camargo AP, Coutinho FH, et al. iPHoP: An integrated machine learning framework to maximize host prediction for metagenome-derived viruses of archaea and bacteria. *PLOS Biol* 2023;**21**:e3002083. https://doi.org/10.1371/journal.pbio.3002083

65.	Müller C, Zhang L, Zipfel S, et al. Molecular interplay of an assembly machinery for nitrous oxide reductase. *Nature* 2022;**608**:626–631. https://doi.org/10.1038/s41586-022-05015-2

66.	Edgar RC. MUSCLE: multiple sequence alignment with high accuracy and high throughput. *Nucleic Acids Res* 2004;**32**:1792–1797. https://doi.org/10.1093/nar/gkh340

67.	Eddy SR. HMMER: biosequence analysis using profile hidden Markov models. 2007. 2007.

68.	Roux S, Brum JR, Dutilh BE, et al. Ecogenomics and potential biogeochemical impacts of globally abundant ocean viruses. *Nature* 2016;**537**:689–693. https://doi.org/10.1038/nature19366

69.	Li Z, Pan D, Wei G, et al. Deep sea sediments associated with cold seeps are a subsurface reservoir of viral diversity. *ISME J* 2021;**15**:2366–2378. https://doi.org/10.1038/s41396-021-00932-y

70.	Jaffe AL, Bardot C, Le Jeune A-H, et al. Variable impact of geochemical gradients on the functional potential of bacteria, archaea, and phages from the permanently stratified Lac Pavin. *Microbiome* 2023;**11**:14. https://doi.org/10.1186/s40168-022-01416-7

71.	Santos-Medellín C, Blazewicz SJ, Pett-Ridge J, et al. Viral but not bacterial community successional patterns reflect extreme turnover shortly after rewetting dry soils. *Nat Ecol Evol* 2023;**7**:1809–1822. https://doi.org/10.1038/s41559-023-02207-5

72.	Hyatt D, Chen G-L, LoCascio PF, et al. Prodigal: prokaryotic gene recognition and translation initiation site identification. *BMC Bioinformatics* 2010;**11**:119. https://doi.org/10.1186/1471-2105-11-119

73.	Jumper J, Evans R, Pritzel A, et al. Highly accurate protein structure prediction with AlphaFold. *Nature* 2021;**596**:583–589. https://doi.org/10.1038/s41586-021-03819-2

74.	Abramson J, Adler J, Dunger J, et al. Accurate structure prediction of biomolecular interactions with AlphaFold 3. *Nature* 2024;**630**:493–500. https://doi.org/10.1038/s41586-024-07487-w

75.	Goddard TD, Huang CC, Meng EC, et al. UCSF ChimeraX: Meeting modern challenges in visualization and analysis. *Protein Sci* 2018;**27**:14–25. https://doi.org/10.1002/pro.3235

76.	Meng EC, Goddard TD, Pettersen EF, et al. UCSF ChimeraX : Tools for structure building and analysis. *Protein Sci* 2023;**32**:e4792. https://doi.org/10.1002/pro.4792

77.	Pettersen EF, Goddard TD, Huang CC, et al. UCSF ChimeraX : Structure visualization for researchers, educators, and developers. *Protein Sci* 2021;**30**:70–82. https://doi.org/10.1002/pro.3943
